# Skeletal Muscle Expression of Actinin-3 (*ACTN3*) in Relation to Feed Efficiency Phenotype of F_2_ *Bos indicus* - *Bos taurus* Steers

**DOI:** 10.3389/fgene.2022.796038

**Published:** 2022-02-03

**Authors:** Robert N. Vaughn, Kelli J. Kochan, Aline K. Torres, Min Du, David G. Riley, Clare A. Gill, Andy D. Herring, James O. Sanders, Penny K. Riggs

**Affiliations:** ^1^ Department of Animal Science, Texas A&M University, College Station, TX, United States; ^2^ Department of Animal Sciences, Washington State University, Pullman, WA, United States

**Keywords:** bovine, feed efficiency, fiber type, metabolism, gene network, SNP, ACTN3, sustainability

## Abstract

In this study, actinin-3 (*ACTN3*) gene expression was investigated in relation to the feed efficiency phenotype in *Bos indicus* - *Bos taurus* crossbred steers. A measure of relative feed efficiency based on residual feed intake relative to predictions from the NRC beef cattle model was analyzed by the use of a mixed linear model that included sire and family nested within sire as fixed effects and age, animal type, sex, condition, and breed as random effects for 173 F_2_ Nellore-Angus steers. Based on these residual intake observations, individuals were ranked from most efficient to least efficient. Skeletal muscle samples were analyzed from 54 steers in three groups of 18 (high efficiency, low efficiency, and a statistically average group). *ACTN3*, which encodes a muscle-specific structural protein, was previously identified as a candidate gene from a microarray analysis of RNA extracted from muscle samples obtained from a subset of steers from each of these three efficiency groups. The expression of *ACTN3* was evaluated by quantitative reverse transcriptase PCR analysis. The expression of *ACTN3* in skeletal muscle was 1.6-fold greater in the inefficient steer group than in the efficient group (*p* = 0.007). In addition to expression measurements, blocks of SNP haplotypes were assessed for breed or parent of origin effects. A maternal effect was observed for *ACTN3* inheritance, indicating that a maternal *B. indicus* block conferred improved residual feed efficiency relative to the *B. taurus* copy (*p* = 0.03). A SNP haplotype analysis was also conducted for m-calpain (*CAPN2*) and fibronectin 1 (*FN1*), and a significant breed effect was observed for both genes, with *B. indicus* and *B. taurus* alleles each conferring favorable efficiency when inherited maternally (*p* = 0.03 and *p* = 0.04). Because the *ACTN3* structural protein is specific to fast-twitch (type II) muscle fibers and not present in slow-twitch muscle fibers (type I), muscle samples used for expression analysis were also assayed for fiber type ratio (type II/type I). Inefficient animals had a fast fiber type ratio 1.8-fold greater than the efficient animals (*p* = 0.027). Because these fiber-types exhibit different metabolic profiles, we hypothesize that animals with a greater proportion of fast-twitch muscle fibers are also less feed efficient.

## Introduction

Efficient use of resources is regarded as a critical area of emphasis for livestock production (Kenny et al., 2018; Brito et al., 2021). Feed costs can contribute to 70% of total livestock production costs (Becker 2008). Environmental costs associated with beef production have become increasingly important to the consumer. Although the beef industry made substantial strides in reducing the environmental footprint of cattle production over several decades (Capper 2011), feed efficiency remains a target today in beef producers’ efforts toward sustainability. In considering nutrient security for humans, utilizing new and emerging technologies to better understand the complex physiological mechanisms of production traits will be key for enabling even greater efficiency of food animal production (Riggs et al., 2017; Riggs et al., 2018). Increased access to nutrient-dense animal source foods requires greater production volume if global population growth reaches 9.8 billion people in the coming decades (FAO, 2018). Additionally, a systematic review of literature that examined life cycle assessment in cattle (published between 2000 and 2016) contained climate change as a category, with nearly a third of those papers focusing on only that topic (McClelland et al., 2018). Understanding feed efficiency in the context of breeds adapted to specific environmental conditions—particularly hot, dry conditions found in much of the world’s grazing lands—has important economic and environmental implications.

Brunes et al. (2021) noted that feed efficiency traits in beef cattle are highly complex and affected by numerous biological and polygenic mechanisms. In their GWAS analysis of feed efficiency from 4,329 Nellore cattle, multiple genomic regions were identified that harbor sequence variation associated with greater than 0.5% of the additive genetic variance for feed efficiency in the population (Brunes et al., 2021). Genes within the identified regions contribute to the metabolism of protein, lipids, and numerous metabolic, energetic, and behavioral pathways. A study of dairy cattle in New Zealand (Puillet et al., 2021) examined gene-by-environment interactions. As might be expected, this work demonstrated that the relationships between feed efficiency and other production traits are complex and must be carefully balanced when one considers overall measures of lifetime production efficiencies.

In the present study, we focused on aspects of feed efficiency influenced by skeletal muscle by specifically examining *ACTN3* within a genetic mapping herd of beef cattle that utilized *Bos indicus*–*Bos taurus* cattle suited to the subtropical environment of Texas. Resting energy consumption contributes to feed efficiency as a major component of an animal’s overall energy requirements. Skeletal muscle has lower energy requirements by weight compared to other tissue types. However, because skeletal muscle mass contributes as much as half of the live animal’s weight, a great deal of an animal’s total caloric intake may be utilized for the growth and maintenance of this tissue. Approximately 20% of an animal’s overall energy consumption is consumed by the skeletal muscle (Ortigues 1991), and this percentage can increase drastically during stress, activity, or changes in temperature.

We evaluated *ACTN3* sequence haplotypes and gene expression in F_2_ Nellore-Angus steers that had been evaluated for efficiency. For the study we describe here, we specifically wanted to investigate whether different *ACTN3* alleles in this population corresponded to feed efficiency rates. This gene was identified as one candidate contributing to a feed efficiency phenotype from a pilot gene expression network analysis, and we suspected that animals differed in muscle turnover rate. We hypothesized that we could identify genetic variation in *ACTN3* responsible for differences in muscle physiology and homeostasis that are important for overall feed efficiency. In pigs, a relationship between muscle turnover and residual feed intake was previously described (Cruzen et al., 2013). In beef cattle, the negative correlation of muscle proteolysis rate and feed efficiency has been noted (McDonagh et al., 2001). Other investigators have evaluated skeletal muscle gene expression in beef cattle feed efficiency studies. For example, Lam et al., 2020 developed an RNA sequencing pipeline from liver and muscle transcriptomes (Tizioto et al., 2016) to identify single nucleotide polymorphisms (SNP) associated with feed efficiency in Nellore steers classified with high or low residual feed intake (RFI). In this study, the authors suggested relationships between feed efficiency and both immune and metabolic function in livestock. Regulation of the *RAC1* gene was associated with less efficient cattle. Interestingly, the genetic pathways that include *RAC1* and *ACTN3* may have related functional roles associated with calcium metabolism-based regulation of immune function (Calender et al., 2020).

Also contributing to energetic requirements, different skeletal muscle fiber types have different energy requirements. Type II, or fast-twitch fibers, have greater energy demands than type I, or slow-twitch fibers. A study in horses identified ACTN3 as a potential element associated with muscle performance (Ropka-Molik et al., 2019). Welch et al., 2013 found a positive correlation between relative feed intake (RFI) and type II muscle fibers. This finding is consistent with the idea that the physiological skeletal muscle phenotype of an animal contributes to its overall efficiency phenotype. Because ACTN3 is expressed only in type II fibers, this study also examined fiber type profiles in muscle samples.

## Materials and Methods

### Feed Efficiency Phenotype

For this study, feed and carcass data, obtained from 173 Nellore-Angus F_2_ steers in 13 full-sibling families produced by embryo transfer from the Texas A&M McGregor Genomics research population located in central Texas (latitude: 31.3865, longitude: −97.4105), were utilized, as described by Amen (2007). Briefly, calves were produced in the spring and fall calving seasons, and this study used calves born from 2003 to 2005. After weaning (approximately 230 days of age), the animals were grass-fed for approximately 130 days until they reached 11–13 months of age. Steers were moved to a Calan Broadbent Feeding System (American Calan, Northwood, NH, USA) to measure individual feed intake. Over 28 days, the steers were adjusted to the finishing diet (Table 1). Feeding was ad libitum, and uneaten feed was removed and measured every 7 days. Animals were weighed every 28 days for ∼140 days at the same time of day and in the same order of pens at each weigh day to equalize gut fill effects across time, as much as possible.

**TABLE 1:** Ration formulation used for steers in study.

Ingredient	%
Ground milo	20.00
Ground corn	31.25
Cottonseed meal	9.00
Cottonseed hulls	25.00
Molasses	10.00
Premix^a^	3.00
Ammonium chloride	0.25
R-1500^b^	1.50

Ingredients are represented as a percent on an as-fed basis.

a Composition of premix: ground limestone, 60%; trace mineralized salt, 16.7% (NaCl, 98%; Zn, 0.35%; Mn, 0.28%; Fe, 0.175%, Cu, 0.035%, I, 0.007%, Co, 0.007%); mono-dicalcium phosphate, 13%; potassium chloride 6.7%; vitamin premix, 3.3% (vitamin A, 2,200,000 IU/kg; vitamin D, 1,100,000 IU/kg, vitamin E, 2,200 IU/kg); zinc oxide, 0.33%.

b R-1500 contains 1.65 g monensin sodium (Rumensin^TM^) per kg.

Using the NRC (2000) model application, the daily feed intake required to achieve observed ADG was predicted. Model inputs included feeding period mid-weight (used as the current reference weight for model predictions) and final weight at slaughter for each animal. Standardized inputs were used for animal type (growing/finishing), age (14 months), sex (steer), BCS (5), breed (Nellore-Angus two-way cross), management (ionophore), diet (Table 1), and quality grade (select). Environmental factors in the model were set to be thermoneutral. The model predicted intake was subtracted from the observed dry matter intake (DMI), and the difference is defined as residual feed intake based on the NRC model (RFI_NRC_), such that those animals that consumed less than predicted (thus, were more efficient) had negative RFI_NRC_ (Liu et al., 2000). Mixed procedures of SAS were then used to analyze RFI_NRC_ with fixed factors of sire and family nested within sire (Amen 2007). This method of residual prediction parallels more typical calculations of RFI, in which individual residuals represent a deviation from a modeled mean intake at an observed rate of gain. However, the standard method is generally restricted to use within a single contemporary group fed simultaneously or with the use of the contemporary group as a model effect. Importantly, in this case, a standard model (the NRC) was utilized to construct the modeled mean intake rather than a regression on observed data, thus enabling animals in multiple contemporary groups to be evaluated against a common model.

### Sample Selection

For gene expression analysis, 36 animals were identified at the tails of the efficiency distribution based on RFI_NRC_ as described above. A total of 18 animals were classified as most “efficient” for this population and had negative RFI_NRC_ residuals indicating that they had consumed less feed than would be expected based on the model. A total of 18 animals were classified as most “inefficient,” with positive RFI_NRC_ residuals indicating they had consumed more feed than would be expected based on the model. Muscle samples from these 36 animals were used for subsequent expression analysis. In addition, a statistically average group of 18 animals with an RFI_NRC_ residual clustered around zero was included for comparison purposes. Thus, a total of 54 animals from the middle, and both tails of the residual distribution were analyzed for gene expression. Means for these groups are presented in Table 2. The distribution of these groups across birth year seasons (BYS) is shown in Table 3. In addition, the frequency distribution of animals from the Amen (2007) study is presented in Table 4, with the set of most and least efficient animals shown in parentheses. Amen (2007) noted that imbalance exists among families across BYS. While this imbalance may complicate efforts to separate family effects from year-season effects, the approach provides a means to examine the combined overall impact of gene X environmental interactions on resultant phenotypes to understand the influence of gene function on phenotype despite a range of regional climate conditions.

**TABLE 2:** Simple means (±STD err) for RFI_NRC_ residuals by efficiency groups.

Item	Efficient			Average			Inefficient		
*n*	18			18			18		
RFI_NRC_ residuals	−2.3	±	0.1^a^	0.0	±	0.0^b^	2.3	±	0.1^c^

a Means within a row with different superscripts differ significantly *(p* < 0.01).

b Means within a row with different superscripts differ significantly *(p* < 0.01).

c Means within a row with different superscripts differ significantly *(p* < 0.01).

**TABLE 3:** Birth year season (BYS) distribution among efficiency groups.

Group	*N*	F03	F04	F05	S03	S04	S05
Efficient	18	0	1	0	1	2	14
Average	18	3	4	4	1	4	2
Inefficient	18	5	4	6	1	2	0

Season is designated as Fall (F) or Spring (S).

**TABLE 4:** Frequency table of family distribution across BYS contemporary groups from animals evaluated for feed efficiency in a previous study (Amen, 2007).

Contemporary group							
Family ID^a^	S2003	F2003	S2004	F2004	S2005	F2005	Total
70	1	5	4 (E 1)	2 (I 1)	1 (E 1)	4	17
71	2	2	5	4	2 (E 2)	0	15
72	4 (E 1)	0	5 (I 1)	0	2 (E 2)	7	18
73	2	3	0	0	0	0	5
74	4	0	0	0	0	0	4
75	5 (I 1)	0	0	2	4 (E 1)	0	11
76	2	3	0	0	0	0	5
77	1	5 (I 5)	1 (I 1)	1	9 (E 3)	0	17
80	0	7	3 (E 1)	16 (I 1)	0	1	27
81	0	1	11	3	5 (E 3)	5 (I 3)	25
82	0	0	0	0	0	6	6
83	0	0	3	2	4 (E 2). (I 1)	2 (I 2)	11
84	0	0	0	1	7 (E 1)	4 (I 1)	12
Total	21	26	32	31	34	29	173

Season is designated as Fall (F) or Spring (S). Distribution of animals identified in the group of 18 most efficient (E-n) or 18 most inefficient (I-n) evaluated in the current study is identified in parentheses.

a Sire ID and respective families 297J–70, 71; 432H–72, 73; 437J–74, 75, 81, 82, 83; 551G–76, 77, 80, 84. Although not all families are represented in all contemporary groups, sires are much more evenly distributed across these groups. All sires had at least three steers per contemporary group except for 432H in Fall 2004 (*n* = 0) and Spring 2005 (*n* = 2) and 437J in Fall 2003 (*n* = 1).

### Tissue Collection and Extraction of RNA

Steers were harvested at 18 months of age at the Rosenthal Meat Center at Texas A&M University in College Station, TX using humane harvesting procedures, as described by Savell and Smith (2000). Animals were restricted from feed for approximately 12 h before harvest but had continual access to water. Animals were immobilized using a captive bolt stunning mechanism and further processed using standard industry procedures. Approximately 1 g of muscle tissue from the *Longissimus cervicis* (in the neck region of the carcass) was collected before electrical stimulation (ES; less than 1 h after exsanguination) of the carcass. The muscle sample was flash-frozen in liquid nitrogen to prevent mRNA degradation. Samples were stored at −80°C until RNA was extracted.

Total RNA was extracted from a portion (∼100–200 mg) of the frozen whole muscle tissue samples (*L. cervicis*) from each of the 54 animals with TRI Reagent^®^ (Molecular Research Center, Cincinnati, OH, USA) and 1-bromo-3-chloropropane (Molecular Research Center). Next, RNA was precipitated with isopropanol (Sigma Aldrich, St. Louis, MO, USA), washed with 70% ethanol (Sigma Aldrich), and reconstituted in 50 µl nuclease-free water (Invitrogen, Carlsbad, CA, USA). The quality of the RNA was assessed *via* an Agilent 2100 series Bioanalyzer (Agilent Technologies, Palo Alto, CA, USA) according to the manufacturer’s protocol. Samples with an RNA integrity number (RIN) >8.0 and appropriate electropherogram image were treated with DNase (Invitrogen) and column-purified *via* the RNeasy Mini kit (Qiagen, Valencia, CA, USA). Samples were quantified by spectrophotometry (NanoDrop 1000), and identical quantities of total RNA for each sample were utilized in downstream applications. RNA extracts were stored at −80°C prior to expression analyses.

### Microarray Procedures

Microarray analysis (described in Vaughn, 2013; Riggs and Vaughn, 2015) was conducted prior to this study as a pilot study with the Agilent 44k bovine array (*B. taurus* oligo microarray V2 Agilent 4x44k GPL11649) according to manufacturer’s recommendations. Twenty-four RNA samples (200 ng each from eight most efficient and eight least efficient animals) were labeled for two-color microarray gene expression analysis (Quick Amp Labeling Kit, Agilent Technologies) according to the manufacturer’s protocol. Following hybridization and scanning, normalization and quality control were performed using embedded quantile normalization functions in the Genespring GX v11.0.2 software. Following quality filtering, the Mann–Whitney unpaired test was utilized. A non-parametric test was used to avoid relying on the *a priori* assumption of a normal distribution of gene expression within these populations selected for extremes. In this experimental design, samples fail the assumption of independence, making false discovery analyses invalid. A cut-off of *p* ≤ 0.05 and a fold-difference of 1.4-fold or greater were set as thresholds to generate lists of probes for subsequent pathways analysis. Pathway analysis was performed using DAVID Bioinformatics Resources 6.7 (http://david.ncifcrf.gov/). Gene Ontology analysis was also performed within the DAVID software using the same data set used for pathway analysis. The GO-fat category was used with the default ease setting of 0.1 and a minimum count of 2. From these pathways analyses, as well as a separate and independent array and pathway experiments (not described here), muscle metabolic pathways we formed a hypothesis related to genes involved in skeletal muscle turnover. As a result, *ACTN3* was identified as a target for further investigation by qRT-PCR and haplotype analysis. Array data are deposited in the Gene Expression Omnibus, GEO accession number GSE56705 https://www.ncbi.nlm.nih.gov/geo/query/acc.cgi?acc=GSE56705.

### Real Time Quantitative Reverse Transcriptase Polymerase Chain Reaction

Quantitative RT-PCR analysis was conducted on the full set of 54 samples. Synthesis of cDNA from all RNA samples with the high-capacity cDNA Reverse Transcription Kit (Invitrogen) was accomplished with a starting quantity of 2 µg mRNA per 40 μl reaction. Oligo(dT)20 primers (Integrated DNA Technologies, Coralville, IA, USA), 5 μM final concentration, were used for reverse transcription. cDNA was diluted 1:2 in yeast tRNA (25 ng/μl; Invitrogen). Samples were amplified in a total volume of 20 µl containing 1X SYBR GreenER^™^ qPCR SuperMix (Invitrogen), 300 nM primers and 2 μl template cDNA. Real-time quantitative RT-PCR (qRT-PCR) was performed at 95°C for 10 min followed by 40 cycles of 15 s at 95°C and 60 s at 60°C, in a 7900HT thermal cycler (Applied Biosystems).

Primers for genes validated by qRT-PCR were designed within Oligo 6 Primer Analysis Software v6.71 (Molecular Biology Insights, Cascade, CO, USA). The analyzed genes included actinin-2 (*ACTN2*), actinin-3 (*ACTN3*), adipose differentiation-related protein (*ADFP*, also known as perilipin 2 (*PLIN2)*), ATP synthase H+ transporting, mitochondrial F1 complex, beta polypeptide (*ATP5B*), hexokinase 2 (*HKII*), and lactate dehydrogenase B (*LDHB*). Ribosomal protein S20 (*RPS20*) was utilized as a reference gene. Genes and primers used are described in Table 5. Primer pairs were evaluated by BLAST sequence similarity search (Altschul et al., 1990). Primer pairs were selected, which did not cross-amplify across species or between different mRNA transcripts. Additionally, primers were selected to span an exon junction to prevent genomic amplification.

**TABLE 5:** Complete list of primer pairs used for qRT-PCR assays and their function in muscle.

Gene symbol	Description	Sequence^a^
*ACTN2*	Actinin, alpha 2	5ʹ-GGT​CTT​TGA​CAA​CAA​GCA-3ʹ
		5ʹ-TGA​TGG​TTC​TGG​CGA​TA-3ʹ
*ACTN3*	Actinin, alpha 3	5ʹ-CGG​GAG​ACA​AGA​ACT​ACA​TCA-3ʹ
		5ʹ-CGT​AGA​GGG​CAC​TGG​AGA​A-3ʹ
*ATP5B*	ATP synthase, H+ transporting	5ʹ-CCC​ATC​AAA​ACC​AAG​CAA-3ʹ
	Mitochondrial F1 complex	5ʹ-TCA​ACA​CTC​ATC​TCC​ACG​AA-3ʹ
	Beta polypeptide	
*CAPN1*	Calpain 1, (mu/I) large subunit	5ʹ-GAC​CAT​AGG​CTT​CGC​TGT​CT-3ʹ
		5ʹ-AGG​TTG​ATG​AAC​TGC​TCG​GA-3ʹ
*CAPN2*	Calpain 2, (m/II) large subunit	5ʹ-CGA​CTG​GAG​ACA​CTG​TTC​AGG​A-3ʹ
		5ʹ-CTT​CAG​GCA​GAT​TGG​TTA​TCA​CTT-3ʹ
*CAST*	Calpastatin	5ʹ-GCT​GTC​GTC​TCT​GAA​GTG​GTT-3ʹ
		5ʹ-GGC​ATC​GTC​AAG​TTC​TTT​GTT​GT-3ʹ
*HKII*	Hexokinase 2	5ʹ-TCA​ACA​CTC​ATC​TCC​ACG​AA-3ʹ
		5ʹ-CAC​CAC​AGC​AAC​CAC​ATC-3ʹ
*LDHB*	Lactate dehydrogenase B	5ʹ-CAG​AAA​TGG​GAA​CAG​ACA​A-3ʹ
		5ʹ-GAC​TTC​ATA​GGC​ACT​CTC​AAC-3ʹ
*MYH1*	Myosin, heavy chain 1, skeletal muscle, adult	5ʹ-TGA​GGA​AGC​GGA​GGA​ACA​AT-3ʹ
		5ʹ-TGG​GAC​TCG​GCA​ATG​TCA-3ʹ
*MYH2*	Heavy chain 2, skeletal muscle, adult	5ʹ-CAA​TGA​CCT​GAC​AAC​CCA​GA-3ʹ
		5ʹ-CCT​TGA​CAA​CTG​AGA​CAC​CAG​A-3ʹ
*RPS20*	Ribosomal protein S20	5ʹ-ACC​AGC​CGC​AAC​GTG​AA-3ʹ
		5ʹ-CCT​TCG​CGC​CTC​TGA​TCA-3ʹ

a *RPS20* primer sequences provided by Kochan et al. (2009).

To quantify qRT-PCR results, amplification data were analyzed *via* Sequence Detection System v2.4 software (Applied Biosystems, Carlsbad, CA, USA). Expression was normalized to RPS20 as a reference gene (De Jonge et al., 2007), and relative expression was quantified as described by Livak and Schmittgen (2001). A threshold value of 0.20 was used to determine the C_t_ value. In summary, raw C_t_ values were normalized to *RPS20* based on internal expression stability between groups (Vandesompele et al., 2002). The normalized value was subtracted from the raw C_t_ for each sample (Δ C_t_). From the Δ C_t_, the average value of the efficient group was subtracted (ΔΔ C_t_). The ΔΔ C_t_ value was linearized by conversion to the inverse negative log_2_ (RQ). The efficient group in this study thus has a median expression value of 1.0. It should be noted that these are arbitrary units (AU) of expression and that no direct comparison between different genes in total expression levels can be reliably made. All values are relative and applicable directly only as a within-group comparison of relative expression.

### Muscle Fiber Type Classification

Fiber type analysis was determined by gel electrophoresis. *L. cervicis* muscle samples (0.1 g) were homogenized in 500 μl buffer consisting of 250 mM sucrose, 100 mM KCl, 5 mM EDTA, and 20 mM Tris-HCl pH 6.8. The homogenate was filtered through nylon cloth to remove debris and centrifuged at 10,000 × *g* for 10 min. The pellet obtained was resuspended in 500 μl washing buffer (200 mM KCl, 5 mM EDTA, 0.5% Triton X-100, and 20 mM Tris-HCl pH 6.8). The suspension was centrifuged at 10,000 × *g* for 10 min. The pellet containing purified myofibrillar proteins was resuspended in 200 μl water and 300 μl of standard 2X sample loading buffer, boiled for 5 min, and then centrifuged at 12,000 × *g* for 5 min. The resultant supernatant was used for electrophoresis.

Stacking gels consisted of 4% acrylamide (acrylamide: bis = 50:1) and 5% (v/v) glycerol, 70 mM Tris-HCl pH 6.7, 0.4% (w/v) SDS, 4 mM EDTA, 0.1% (w/v) APS, and 0.01% (v/v) TEMED. The separation gel contained 5% (w/v) glycerol, acrylamide: bis (50:1) at a concentration ranging from 5 to 20%, 200 mM Tris pH 8.8, 4 mM EDTA, 0.4% (w/v) SDS, 0.01% (v/v) TEMED, and 0.1% (w/v) ammonium persulfate. The upper running buffer consisted of 0.1 M Tris-HCl pH 8.8, 0.1% (w/v) SDS, 150 mM glycine, and 10 mM mercaptoethanol, and the lower running buffer consisted of 50 mM Tris-HCl pH 8.8, 0.01% (w/v) SDS, and 75 mM glycine. Gels were run at 8°C in a Hoefer SE 600 (Hoefer Scientific, San Francisco, CA, USA) unit, at constant 200 V for 24 h (Bamman et al., 1999). After electrophoresis, gels were stained with Coomassie blue and scanned with a densitometer to determine the amount of each myosin isoform and the percentage of type I and type II muscle fibers was reported (Underwood et al., 2007).

### Haplotype Analysis

All individuals (*n* = 776) from the first three generations of the experimental population were previously genotyped with the Illumina BovSNP50 v1 assay (Illumina, Inc., San Diego, CA, USA), and data were available for use in this study. The haplotype analysis from these data allowed us to trace the inheritance of Nellore or Angus blocks of SNPs and identify the parent of origin for the 173 Nellore-Angus F_2_ steers for which RFI_NRC_ data and tissue samples were collected and used in this study. After pruning genotypes to remove animals with poor completion rate (<0.9), SNP with low minor allele frequency (<0.05), SNP with poor completion rates (<0.9), and those SNP that deviated from Hardy–Weinberg equilibrium (*p* < 0.0001), 39,890 genotypes per individual remained. To determine whether breed of origin or parent of origin played a role in the efficiency phenotype, SNP genotypes spanning 1 Mb intervals flanking several genes of interest (Table 6) based on expression analyses were extracted using PLINK v1.07 (Purcell et al., 2007) and formatted for phase v2.1.1 software (Stephens et al., 2001). Haplotypes were established using 100 iterations of phase v2.1.1, with a thinning interval of 2 and a burn-in of 10. Resultant phased haplotypes were ordered by generation, and breed (Nellore or Angus) and parent of origin were manually tracked through the pedigree to assign the source of each haplotype block in the F2 generation. Because gene expression analyses of μ-calpain (*CAPN1*), m-calpain (*CAPN2*), calpastatin (*CAST*), myosin heavy chain 1 (*MYH1*), and myosin heavy chain 2 (*MYH2*) genes were also available from another study and are relevant to pathways associated with muscle growth and proteolysis (Vaughn 2013), their relationship with feed efficiency was also examined. The *ACTN2* gene was selected for analysis because it is expressed in all skeletal muscle fiber types and has conserved structural and functional similarity to *ACTN3*. The genes *CAPN1*, *CAPN2*, and *CAST* were examined because of their roles in muscle proteolysis (Goll et al., 2003), and MYH1 and MYH2 were selected for their function in muscle fibers (Wang et al., 2012).

**TABLE 6:** SNP haplotype block location information.

Gene	Chromosome	Coordinate (UMD 3.1)		Number of SNPs in 1 Mb region
*CAPN1*	29	44063463	44113492	24
*CAPN2*	16	27781671	27840011	24
*CAPN3*	10	37829007	37885645	24
*CAST*	7	98444979	98581253	18
*ACTN2*	28	9403203	9450920	16
*ACTN3*	29	45230630	45242406	16
*MYH1/2*	19	30110728	30165109	18
*FN1*	2	103881402	103950562	12

Locations according to *Bos taurus* UMD 3.1 genome assembly.

### Statistical Analysis

SPSS 16.0 software (IBM, Armonk, NY) was used for all statistical analyses. To test for significance between efficiency groups, an independent samples *t*-test was used. Correlation analysis used a bivariate two-tailed Pearson’s correlation. All qRT-PCR expression was normalized to the *RPS20* reference gene (De Jonge et al., 2007). Expression data are reported relative to the feed efficient group, which was set to a value of 1.0. The inefficient and average groups are represented as fold difference relative to the efficient group. One sample was removed from all analyses because expression values measured for the reference gene, *RPS20*, were not consistent with other samples.

## Results

### Gene Expression Analysis

Following an initial pilot microarray (GEO accession number GSE56705) and network pathway analysis, the *ACTN3* gene was selected for further investigation. From the initial hypothesis generating microarray experiment, 58 genes were expressed significantly differently between efficiency groups with a fold difference between groups of 1.5-fold or greater (Supplementary Table S1). The *ACTN3* gene was expressed as 2.5-fold greater in the inefficient group of steers but fell short of significance (*p* = 0.051). However, because different alleles of this gene have been associated with differential athletic performance in humans, and *ACTN3* genotype is thought to contribute to variation in muscle phenotype (North et al., 2003; Yang et al., 2003), *ACTN3* was selected for further investigation. Despite missing the initial statistical cut-off, *ACTN3* also remained of interest because it is a member of skeletal muscle networks determined to be relevant. Following these early investigations, this study was developed to examine *ACTN3* as a candidate gene contributing to a feed efficiency phenotype. Genes used for qRT-PCR analysis are described in Table 5. Additional genes that have some relationship to *ACTN3* and the skeletal muscle pathways in which it participates were included in qRT-PCR expression and haplotype analyses. These genes were selected prior to expression and haplotype analysis as negative control genes or as genes expected to function similarly in given pathways. We thought that, by examining haplotypes according to parent of origin, we may be able to differentiate favorable *Bos indicus* and *Bos taurus* alleles that would be of use for selection. The *ACTN2* gene was selected for analysis because it is expressed in all skeletal muscle fiber types and has conserved structural and functional similarity to *ACTN3*. Although their functions are not identical, evidence exists that *ACTN2* can largely compensate for the absence of *ACTN3* (Mills et al., 2001; Lek et al., 2010). The previously evaluated reference genes were tested for stability, and *RPS20* was utilized as a control reference gene. Because of our hypothesis that *ACTN3* was associated with feed efficiency and might reflect differences in overall metabolic activity, we also selected a set of genes that are known to play a role in other metabolic processes within muscle tissue to examine at the same time. For example, *ADFP* was selected because of its role in lipid metabolism and storage (Hiller et al., 2012). Genes that place a role in muscle metabolism and glucose homeostasis (*ATP5B*, *HKII*, and *LDHB*) were also examined because these are genes that play a role in muscle metabolism (Rajesh et al., 2011; Egan and Zierath 2013; Li et al., 2013). The genes *CAPN1*, *CAPN2*, and *CAST* were examined because of their roles in muscle proteolysis (Goll et al., 2003), and *MYH1* and *MYH2* were selected for their function in muscle fibers (Wang et al., 2012).

By qRT-PCR, expression of *ACTN3* was 1.6-fold greater (*p* = 0.009) in the average and inefficient groups than in the efficient group (Table 7). *ACTN2* expression did not vary significantly between any of the groups, nor did it correlate significantly with *ACTN3* expression or fiber type ratio (as expected). Of the other genes assayed, *LDHB* expression was significantly lower in the average group but not the inefficient group, relative to the efficient steers. The mRNA quantity of the remaining genes examined (Table 7) did not differ significantly between groups.

**TABLE 7:** Relative mRNA expression of selected genes by qRT-PCR analysis.

Gene	Ratio average/efficient			Ratio inefficient/efficient		
*N*	18			18		
*ACTN3*	1.6	±	0.06^a^	1.6	±	0.05^a^
*ACTN2*	1.2	±	0.050	1.0	±	0.050
*COX3*	1.7	±	0.05^a^	1.4	±	0.06^b^
*COX7C*	1.1	±	0.030	0.9	±	0.040
*HKII*	0.9	±	0.040	0.8	±	0.050
*LDHB*	0.6	±	0.03^a^	0.9	±	0.04^b^
*PRDX3*	0.9	±	0.030	1.0	±	0.040

Relative expression for the average and inefficient groups is calculated and presented as a fold ratio compared to the efficient group. Expression was normalized to *RPS20*, and expression value is presented as the geometric mean ratio of arbitrary expression units relative to expression in the efficient group. Each group (average, efficient, inefficient) reflects samples from 18 animals.

a Means with superscripts differ from the efficient group (*p* < 0.05).

b Means with different superscripts differ from each other (*p* < 0.05).

**FIGURE 1:**
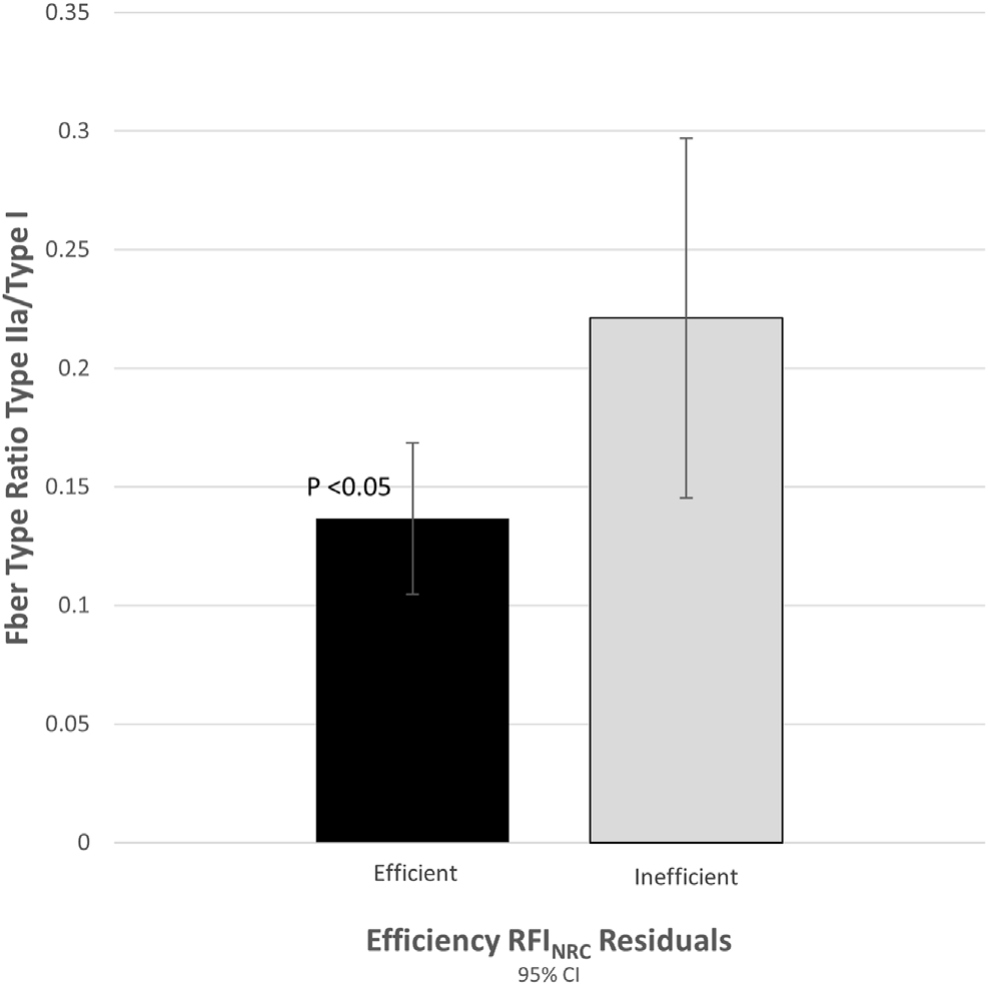
Proportion of fast-to-slow-twitch (type II/type I) fiber ratio was correlated with expression of actinin-3 (*r* = 0.622, *p* < 0.001).

Post-transcriptional modifications, other regulatory mechanisms, and differences in the timing of expression can make the correlation of mRNA expression with protein expression difficult (Greenbaum et al., 2002). To verify that the observed difference in *ACTN3* gene expression translated to actual differences in muscle protein expression, 12 samples (portions of the same samples used for qRT-PCR) from each tail of the distribution (*n* = 24) were assayed for fiber type ratios based on gel separation of muscle fiber type specific isoforms. The ratio of fast-twitch to slow-twitch muscle fiber (type II/type I) was 1.8-fold greater (*p* = 0.027) in the inefficient group compared to the efficient group (Figure 1).

### Haplotype Analysis

Haplotype block analysis was conducted for *ACTN2*, *ACTN3*, *CAPN1*, *CAPN2*, *CAPN3*, *CAST*, *FN1*, and *MYH1/2*. Three of the haplotype blocks were associated with significant differences in efficiency between Angus and Nellore in the larger population studied. The *CAPN2* and *ACTN3* Nellore haplotype blocks were associated with a superior efficiency when inherited maternally (*p* = 0.03). The *FN1* Angus haplotype block was associated with a superior efficiency when inherited maternally (*p* = 0.04). Neither the *CAST* nor the *CAPN1* haplotypes were associated with any improvement in efficiency (Table 8).

**TABLE 8:** RFI residuals based on haplotype block analysis.

		Haplotype block												Paternal						Maternal					
		NN			NA			AN			AA			NN/NA			AN/AA			NN/AN			NA/AA		
	RFI res	−0.7	±	0.5	0.5	±	0.4	−0.3	±	0.4	0.5	±	0.4	0.0	±	0.3	0.1	±	0.3	−0.5^a^	±	0.3	0.5^b^	±	0.3
*CAST*	*n*	46			43			42			43			89			85			88			86		
	RFI res	0.5	±	0.4	−0.8	±	0.4	0.2	±	0.3	0.2	±	0.5	−0.1	±	0.3	0.2	±	0.3	0.4	±	0.3	−0.3	±	0.3
*CAPN1*	*n*	23			39			37			75			62				112	60				114		
	RFI res	−0.6	±	0.6	0.4	±	0.4	−0.3	±	0.4	0.2	±	0.4	0.0	±	0.3	0.0	±	0.3	−0.4	±	0.3	0.3	±	0.3
*CAPN2*	*n*	41			52			40			41			93			81			81			93		
	RFI res	−0.7	±	0.5	0.5	±	0.4	−0.3	±	0.4	0.4	±	0.5	0.0	±	0.3	0.1	±	0.3	−0.5^a^	±	0.3	0.5^b^	±	0.3
*FN1*	*n*	59			52			32			31			111			63			91			83		
	RFI res	0.4	±	0.3	−0.5	±	0.5	0.6	±	0.5	−0.4	±	0.5	0.0	±	0.3	0.1	±	0.3	0.5^a^	±	0.3	−0.4^b^	±	0.3

NN, NA, AN, AA refer to breed haplotype. *N* refers to a haplotype inherited from Nellore, A from Angus. Male is listed first and female second. For example, NA refers to a homozygous animal that has inherited the Nellore haplotype paternally and the Angus haplotype maternally.

a Means within a row with different superscripts differ significantly (*p* < 0.05).

b Means within a row with different superscripts differ significantly (*p* < 0.05).

Season of test feeding period may have influenced efficiency. A general linear model was used to produce the least square means using RFI_NRC_ residuals as the dependent variable with birth year season (BYS) as the fixed factor described in Amen (2007). Six BYS groups were included in this study: F03 (*n* = 26), F04 (*n* = 31), F05 (*n* = 29), S03 (*n* = 21), S04 (*n* = 32), and S05 (*n* = 34), with a total of 87 spring-born animals weaned in the fall and 85 weaned in the spring. Steers weaned in the spring were more efficient than those weaned in the fall; linear contrast of fall RFI_NRC_ residuals means minus spring RFI_NRC_ residuals means was 1.09 + 0.15 (*p* < 0.0001). One BYS level, Spring of 2005, had a lower mean RFI than the other BYS groups (*p* < 0.0001). Within the subset selected for expression analysis, 14 of the 18 samples in the efficient group were from that BYS level (Table 3). The distribution of most and least efficient animals examined is also described in Table 4.

*ACTN3* expression was higher in fall weaned animals than spring weaned animals by 2.1-fold (*p* < 0.001). No significant differences between seasons were noted in any other genes assayed. Additionally, a difference (*p* < 0.001) in fiber type by the season of weaning was observed. Those calves weaned in the fall (*n* = 11) had a fast-to-slow-twitch (type II/type I) fiber type ratio 1.6-fold greater than those weaned in the spring (*n* = 13). These ratios were 0.23 and 0.14, respectively.

## Discussion

Multiple biological and developmental processes, driven by complex genetic networks and response to environmental conditions, each contribute to phenotypic measures of efficiency and the overall relationships of inputs (e.g., feed intake) to outputs (e.g., meat and other products). The identification of specific genetic variation contributes to better understanding and insight into mechanisms of growth and energy utilization. Such knowledge can drive further gains in productivity, reducing waste and environmental impacts. Increased feed efficiency can reduce production costs (Ho et al., 2013) and environmental footprint, both of which are important in a world with an expanding population to feed and finite resources for the production of necessary animal source foods. Waste concerns, from feed waste to methane production, have also gained emphasis in recent years (Brito et al., 2020). Simultaneously, consumers are demanding environmentally conscious options, while producers strive to maintain fiscal viability.

In the present study, a difference in *ACTN3* expression between groups was confirmed by qRT-PCR analysis in skeletal muscle samples from the high and low feed efficiency groups of steers. Expression of *ACTN2* did not differ between groups. Relative to the efficient group, the inefficient group overexpressed *ACTN3* 1.6-fold. The fiber type ratio measured by fast-twitch (type II)/slow-twitch (type I) differed between groups, with a 1.8-fold increase in the inefficient group relative to the efficient group. Season of weaning may have influenced the results since steers weaned in the fall also had a greater fast-twitch to slow-twitch fiber type ratio and greater expression of *ACTN3* compared with those weaned during spring. However, the model predicted RFI_NRC_ does consider birth year season. Feed intake and efficiency have been previously shown to be affected by seasonal effects (Mujibi et al., 2010).

Although not all families are represented in all contemporary groups for which feed efficiency data were collected (Table 4), sires are much more evenly distributed across these groups. All sires had at least three steers per contemporary group except for 432H in Fall 2004 (*n* = 0) and Spring 2005 (*n* = 2) and 437J in Fall 2003 (*n* = 1). Because RFI_NRC_ is a population-based calculation rather than a cohort-based calculation, its use may produce unequal numbers of efficient/inefficient observations per contemporary group. Although only 1 or 0 animals are evaluated from a single contemporary group, this should not bias the results compared to if all animals were included in the analyses.

Our use of RFI_NRC_ enabled animals to be compared across BYS groups. However, as evidenced by the imbalance of animals deemed “most efficient” across BYS, we are likely observing the impact of G X E interaction on the efficiency phenotype. However, despite a potential environmental influence that also affected efficiency, we identified the most efficient animals across all BYS. We are interested in understanding genes and gene networks that contribute to multigenic traits such as feed efficiency in the context of variably subtropical conditions that are typically experienced in beef cattle-producing regions such as Texas.

As *ACTN3* is expressed only in type II fibers, the fiber type data are consistent with our expression data. The inefficient group also had a larger standard error than the efficient group. A possible explanation for this variability in expression could be that an animal may be inefficient, or just simply average, due to a wide array of combined genotypic, environmental, and other factors. In contrast, an efficient animal might be expected to possess only specific genotype combinations that, in conjunction with certain environmental conditions, return an efficient phenotype. Fiber type ratio is also just one variable among many that could possibly reduce overall efficiency.

A role for *ACTN3* variation in growth and metabolism is supported by studies in other species, including humans. A relationship between ACTN3 expression and metabolic rates has been reported in rodents (MacArthur et al., 2007; MacArthur et al., 2008; Ogura et al., 2009) and humans (North and Beggs 1996; North et al., 2003; Yang et al., 2003). Additionally, the relationship between the *ACTN3* R577X (loss of function) polymorphism and elite athletic performance has been described in humans (Roth et al., 2008; Ahmetov et al., 2010; Fiuza-Luces et al., 2011). Interestingly, loss of function has been shown to be associated with the reduced cross-sectional area and thigh muscle volume in older women (Min et al., 2016; Kiuchi et al., 2021).

Among all steers with records, across all seasons in the study (*n* = 173), from which a subset was used for gene expression assays, parent and breed of origin of the *ACTN3* haplotype block had a significant impact on efficiency as measured by RFI residuals. The Nellore (*Bos indicus*) haplotype block was associated with greater efficiency when inherited maternally (RFI residual −0.47 for maternal Nellore inheritance compared to 0.49) regardless of the breed of origin of the paternal haplotype block. If efficiency is represented in terms of feed efficiency during a 180-day feedlot finishing period in kg/d, at current US prices of $0.352/kg, this difference will result in about $63 savings per animal for the most efficient animals. Additionally, the Nellore haplotype blocks of *CAPN2* and *FN1* were associated with differences in efficiency when inherited maternally. For *CAPN2*, a maternally inherited Nellore haplotype was linked to improved efficiency (RFI residual −0.50 compared to 0.50). The *FN1* Angus haplotype block was associated with improved efficiency relative to the Nellore (−0.40 compared to 0.50). No paternal haplotype blocks were implicated to play a role in this trait. These results suggest that parent of origin effects or other epigenetic effects may play an important role in the efficiency phenotype and should be investigated further to optimize management practices. Reciprocal differences in birthweight between *B. indicus* X *B. taurus* and *B. taurus* X *B. indicus* offspring are well known (Dillon et al., 2015). Epigenetic differences in regulation of skeletal muscle growth have also been described, notably in callipyge sheep, where certain transcripts detected in skeletal muscle are transcribed from only a single parental allele (Bidwell et al., 2004). Although feed efficiency data are available only for the F_2_ steers utilized in this study and not the parent/grandparent generations nor sibling heifers, these findings are relevant for beef cattle production strategies in subtropical regions. It will be necessary to understand the impact of these same regions on heifers, replacement cows, and future sires. Additionally, these data begin to contribute to the understanding of parent-of-origin effects on the inheritance of chromosomal regions—particularly important for regions where *Bos taurus*–*Bos indicus* cross cattle are utilized.

Regarding fiber types, fast- and slow-twitch muscle fibers rely primarily on different metabolic pathways. Fast-twitch fibers (type II) rely on the anaerobic degradation of glucose for energy (glycolysis). In contrast, slow fibers (type I) utilize the aerobic citric acid cycle as a primary energy source. Due to the differences in metabolic pathways utilized, a shift in one direction can lead to overall differences in energy utilization during the lifespan of the animal. A reduction in *ACTN3* can result in differences in glycogen phosphorylase activity in muscle and changes in calcium metabolism (MacArthur et al., 2007; Quinlan et al., 2009; Quinlan et al., 2010). Mice entirely deficient in *Actn3* show an increase in expression of enzymes relating to the glycolytic pathway and a decrease in expression of enzymes of the aerobic cellular respiration pathway (MacArthur et al., 2008). Aerobic cellular respiration in total produces 38 molecules of ATP per molecule of glucose input compared to only two molecules of ATP produced by glycolysis per molecule of glucose, making aerobic metabolism 19 times more efficient than glycolysis. Therefore, any shift towards one over the other may affect the overall efficiency of energy metabolism. A greater abundance of *ACTN3* and Type II fibers may contribute mechanistically to seasonal differences.

Cattle facing insufficient nutrition degrade fast-twitch muscle fibers initially to preserve slow-twitch muscle fibers (Lehnert et al., 2006), suggesting a preference for higher efficiency muscle under periods of nutritional stress. To our knowledge, a role for *ACTN3* expression specifically influencing bovine metabolic efficiency has not been previously described. However, Nolte et al., 2019 examined biological networks to identify key long non-coding RNAs (lncRNA) associated with bovine metabolic efficiency. Of three key lncRNAs, one lncRNA with connectivity to low metabolic efficiency (MSTRG.10337) and expressed in muscle tissue was correlated with calcium signaling and other skeletal muscle genes, including *ACTN3*. It is unclear what role *CAPN2* and *FN1* might play in affecting efficiency, but these results are also consistent with the Nolte study of a potential role for calcium and cytoskeletal signaling. Because both *CAPN2* and *FN1* are associated with muscle turnover and growth, it is possible they are linked to differences in the rates of these physiological functions, which would, in turn, alter the metabolic rate of the animal. Interestingly, Karisa et al. (2013) demonstrated the association of *CAST* genes with efficiency. The animals in their study were crossbred *Bos taurus* breeds reared in Canada. Potentially, the favorable *Bos indicus ACNT3*, *CAPN2*, and *FN1* haplotypes reflect the more favorable suitability of these breeds for subtropical environments.

In this experiment, we demonstrated that, in skeletal muscle of Nellore-Angus F_2_ steers, samples from animals classified as feed efficient (relative to RFI_NRC)_ contained fewer *ACTN3* transcripts in comparison to the inefficient group. Similarly, muscle samples from the efficient group possessed fewer type II muscle fibers than the inefficient group. Maternal inheritance of the Nellore *Bos indicus ACTN3* haplotype block conferred the greatest improvement in efficiency. Two additional genes involved in skeletal muscle turnover, *CAPN2* and *FN1*, were also associated with improved efficiency when inherited as maternal *Bos indicus* haplotype blocks. Collectively these data demonstrate that expression of *ACTN3*, the gene network in which it resides, and its regulation, may represent a novel target for improving feed efficiency in cattle, particularly in subtropical environments. While *ACTN3* may reflect just one element of a trait, these data, in connection with other studies, help provide a base for a better understanding of complex biological mechanisms and their interaction with fluctuating climatic conditions.

## Data Availability

The datasets presented in this study can be found in online repositories. The names of the repository/repositories and accession number(s) can be found at: https://www.ncbi.nlm.nih.gov/geo/query/acc.cgi?acc=GSE56705, GSE56705.
