# Supplementary material for: Skeletal Muscle Expression of Actinin-3 (ACTN3) in Relation to Feed Efficiency Phenotype of F2 Bos indicus - Bos taurus Steers
Source: Front Genet. 2022 Feb 3;13:796038. doi: 10.3389/fgene.2022.796038 (PMC8850926; doi:10.3389/fgene.2022.796038)
Supplement: Supplementary file 1 [file DataSheet1.PDF]

Supplemental Table 1. Microarray results of efficiency study.

Name	*P*-value	Fold Difference	Description
BF074284	0.002438129	0.411067837	Unidentified transcripts on BTA10 position 27141957-27141274
CB533071	0.00248171	0.454745667	Unidentified transcripts on BTA19 position 44123200-44122637
AW417048	0.003300728	0.493687051	Unidentified transcripts on BTA8 position 24418273-24418926
XM_582513	0.003619036	2.3038352	Bos taurus similar to Homo sapiens CTD (carboxy-terminal domain, RNApolymerase II, polypeptide A) small phosphatase 2 (CTDSP2)
XM_604268	0.003776336	2.350378	Bos taurus similar to Homo sapiens carbohydrate (N-acetylgalactosamine4-0) sulfotransferase 9 (CHST9)
CB441016	0.004332499	2.288194	Unidentified transcripts
NM_174344	0.006856954	0.560334147	Bos taurus heat shock 70 kD protein 3 (HSPA3)
XM_867100	0.007180372	1.7105138	Bos taurus similar to Homo sapiens homeobox D1 (HOXD1)
AV606331	0.007270947	5.5743012	Bos taurus similar to Homo sapiens synaptotagmin IV (SYT4)
NM_174773	0.007525125	1.5740873	Bos taurus creatine kinase, muscle (CKM)
CB538551	0.008473322	0.482650966	Unidentified transcripts on BTA16 position 51799535-51799051
XM_593936	0.009057753	2.7212133	Bos taurus similar to Homo sapiens Fanconi anemia, complementation group D2 (FANCD2), transcript variant 1
BM030863	0.009157688	1.6751164	Unidentified transcripts
CB447356	0.009659017	1.6947294	Bos taurus similar to Homo sapiens v-maf musculoaponeurotic fibrosarcoma oncogene homolog (avian) (MAF), transcript variant 1

XM_869454	0.010073992	0.625024532	Bos taurus similar to Homo sapiens hypothetical protein MGC72075 (MGC72075)
NM_001034034	0.01112732	1.5185555	Bos taurus glyceraldehyde-phosphate-dehydrogenase (GAPDH)
XM_584289	0.011341611	1.8452076	Bos taurus similar to Homo sapiens B- cell CLL/lymphoma 6 (zinc finger protein 51) (BCL6), transcript variant 1
XM_584950	0.011533578	0.597342198	Bos taurus similar to Homo sapiensSP100 nuclear antigen (SP100)
XM_613093	0.012606364	0.666148714	Bos taurus similar to Homo sapiens cathepsin S (CTSS)
hmm239033	0.012734083	1.7173122	Bos taurus similar to PREDICTED:Homo sapiens KIAA1394 protein (KIAA1394)
hmm169308	0.013345361	0.526357289	Bos taurus similar to Homo sapiens lysozyme (renal amyloidosis) (LYZ)
XM_881933	0.013365543	0.591363573	Bos taurus similar to Homo sapiens Meis1, myeloid ecotropic viral integration site 1 homolog 2 (mouse) (MEIS2), transcript variant g
NM_001035304	0.013654033	1.5201783	Bos taurus similar to Homo sapiensNDRG family member 2 (NDRG2), transcript variant 2
CB171289	0.021109378	0.655714615	Bos taurus similar to Homo sapiens MAD2 mitotic arrest deficient-like 1(yeast) (MAD2L1)
BE667389	0.021219414	1.5548636	Bos taurus similar to Homo sapiens Cbp/p300-interacting transactivator, with Glu/Asp-rich carboxy-terminal domain, 4 (CITED4)
NM_174542	0.023088146	0.63792158	Bos taurus gamma-aminobutyric acid (GABA) A receptor, alpha 3 (GABRA3)
NM_001038117	0.023795828	0.643819239	Bos taurus similar to Homo sapienscoiled-coil domain containing 52 (CCDC52)
XM_867923	0.024332937	0.651389583	Bos taurus similar to Homo sapiens insulin-like growth factor 1 (somatomedin C) (IGF1)
XM_867687	0.02452165	0.463417069	Bos taurus similar to Homo sapiens mesenchymal stem cell protein DSC54 (LOC51334)

XM_591494	0.029233295	0.493118701	Bos taurus similar to Homo sapiens dynactin 6 (DCTN6)
XM_590109	0.030299604	1.5303572	Bos taurus similar to Homo sapiens pyruvate kinase, muscle (PKM2), transcript variant 1
XM_866591	0.03054445	0.626007598	Bos taurus similar to Homo sapiens TAF15 RNA polymerase II, TATA box binding protein (TBP)-associatedfactor, 68kDa (TAF15), transcript variant 1
NM_173940	0.030559804	1.9760805	Bos taurus myxovirus (influenza) resistance 1, (murine homolog) (MX1)
BE237035	0.03241979	1.5313213	Bos taurus similar to Homo sapiens sarcoglycan, delta (35kDa dystrophin-associated glycoprotein) (SGCD), transcript variant 1
XM_611974	0.032505628	0.613734406	PREDICTED: Bos taurus TBC1 (tre-2/USP6, BUB2, cdc16) domain family, member 1 (TBC1D1), partial mRNA.
NM_001034328	0.032541875	1.886564	Bos taurus similar to Homo sapiensodd-skipped related 2 (Drosophila)(OSR2)
EE943676	0.034238223	0.579403817	Unidentified transcripts on BTA29 position 6143082-6144010
NM_173985	0.03428466	0.632295904	Bos taurus allograft inflammatoryfactor 1 (AIF1)
BE757901	0.034657195	1.6637268	Bos taurus similar to Homo sapiens retinoid X receptor, alpha (RXRA)
NM_174094	0.034901526	1.7993807	Bos taurus inhibin, alpha (INHA)
CB468423	0.034947544	0.615525182	Bos taurus similar to Homo sapiens potassium large conductance calcium-activated channel, subfamily M, beta member 4 (KCNMB4)
XM_582508	0.035982803	1.6649619	Bos taurus similar to Homo sapiens timeless homolog (Drosophila) (TIMELESS)
XM_615433	0.036037516	1.5077794	Bos taurus similar to Homo sapiens guanine nucleotide binding protein (Gprotein), beta 5 (GNB5), transcript variant 2

XM_596652	0.03788836	1.9753999	Bos taurus similar to Homo sapiens eEF1A2 binding protein (DKFZp434B1231)
NM_174014	0.039134722	0.665739735	Bos taurus CD69 antigen (p60, early T-cell activation antigen) (CD69)
XM_865690	0.039325573	0.632320213	Bos taurus similar to Homo sapiens V-set and immunoglobulin domain containing 4 (VSIG4)
XM_613380	0.04393188	0.633518447	Bos taurus similar to Homo sapiens CD163 molecule (CD163), transcriptvariant 1
XM_598942	0.04416452	1.6375349	Bos taurus similar to Homo sapiensleucine rich repeat neuronal 6A (LRRN6A)
NM_001038158	0.04428652	1.5530976	Bos taurus similar to Homo sapiens myeloid leukemia factor 1 (MLF1)
NM_001038579	0.045017164	0.605639191	Bos taurus similar to Homo sapiens family with sequence similarity 33, member A (FAM33A)
BF654881	0.045293685	1.7651677	Bos taurus similar to Homo sapiensneural precursor cell expressed, developmentally down-regulated 4 (NEDD4), transcript variant 2
AV592606	0.0456444	1.5717231	Unidentified transcripts on BTA1 position 77843182-77842501
AW307950	0.045654632	1.6655412	Bos taurus similar to Homo sapiens serine/arginine repetitive matrix 1 (SRRM1)
XM_587120	0.04637955	1.643375	Bos taurus similar to Homo sapiensring finger protein 122 (RNF122)
XM_870939	0.046514437	0.52850603	Bos taurus similar to Homo sapiens sphingosine kinase 1 (SPHK1), transcript variant 1
NM_174210	0.047074508	2.9206915	Bos taurus uncoupling protein 3 (mitochondrial, proton carrier) (UCP3)
XM_581386	0.047203157	1.5165434	Bos taurus similar to Homo sapiens cysteine and histidine-rich domain (CHORD)-containing 1 (CHORDC1)
XM_868860	0.047838625	0.494573539	Bos taurus similar to Homo sapiens cytokine inducible SH2-containing protein (CISH)
XM_882229	0.051343303	2.5111191	Bos taurus similar to Homo sapiens actinin, alpha 3 (ACTN3)

Fold difference is given as inefficient group divided by efficient group.
